# Clinical Manifestations and Risk Factors Associated with 14 Deaths following Swarm Wasp Stings in a Chinese Tertiary Grade A General Hospital: A Retrospective Database Analysis Study

**DOI:** 10.3390/jcm12185789

**Published:** 2023-09-06

**Authors:** Maohe Wang, Mei Qin, Amanda Y. Wang, Jia-Wei Zhao, Fei Deng, Yumei Han, Wei Wang

**Affiliations:** 1 Department of Nephrology, Suining Central Hospital, Suining 629000, China; 2 Renal Department and Nephrology Institute, Sichuan Provincial People’s Hospital, School of Medicine, University of Electronic Science and Technology of China, Chengdu 610072, China; 3 Renal and Metabolic Division, The George Institute for Global Health, UNSW Australia, Sydney, NSW 2052, Australia; 4 Department of Renal Medicine, Concord Repatriation General Hospital, Concord Clinical School, University of Sydney, Camperdown, NSW 2050, Australia; 5 Faculty of Health Sciences and Medicine, Bond University, Gold Coast, QLD 4226, Australia

**Keywords:** wasp, poisoning severity score, death, risk factors, prognosis

## Abstract

Introduction: The objective was to evaluate the poisoning severity score (PSS) as an early prognostic predictor in patients with wasp stings and identify associated clinical characteristics and risk factors for mortality. Methods: A total of 363 patients with wasp stings at Suining Central Hospital between January 2016 and December 2018 were enrolled. Within the first 24 h of admission, the poisoning severity score (PSS) and the Chinese expert consensus on standardized diagnosis and treatment of wasp stings (CECC) were utilized for severity classification, and their correlation was examined. Patients were then divided into survival and death groups based on discharge status. Logistic regression analysis was employed to analyze factors influencing patients’ outcomes. Results: The mortality of wasp sting patients was 3.9%. The PSS and CECC were found to correlate for severity classification. Additionally, female gender, age, number of stings, and PSS were identified as independent risk factors for mortality in wasp sting patients. Combining these four factors yielded an AUC of 0.962 for predicting death. Conclusions: PSS aids in early severity classification of wasp stings. Female gender, age, number of stings, and PSS were independent mortality risk factors in these patients.

## 1. Introduction

The wasps belong to the order Hymenoptera [1,2]. There are more than 6000 species of wasps in the world, with more than 200 species of wasps recorded in China [3]. In Asian countries such as China and Thailand, wasps are the main species responsible for severe clinical symptoms, while carpenter bees or hornets are rarely reported to cause severe clinical symptoms [4,5]. The difference between these two families is easy to recognize. For example, bee or hornet stings usually stay on the victim’s skin, while wasp stings do not [2]. Wasps attack humans in self-defense, and when threatened, they emit warning calls or release a special odor that attracts other wasps to attack. Their venom can cause severe health problems in human beings [6,7].

China is a large agricultural country. With developments in returning farmland to forests in rural areas, vegetation is becoming more and more abundant, providing a suitable habitat for wasps; thus, the incidence of wasp stings is increasing, bringing a serious burden to people’s public health and social economy [5,8]. One or two stings usually result in a mild local reaction, including redness, pain, swelling, rash, or anaphylaxis. A swarm attack can lead to serious systemic toxicity, such as rhabdomyolysis, hemolysis, ARDS (acute respiratory distress syndrome), AKI (acute kidney injury) and death [9,10,11]. The mortality after wasp stings ranged from 5.1 to 21% [5]. It is particularly important to classify the severity at an early stage and to carry out the corresponding treatment [12,13]. The severity of wasp stings is related to the species and the number of stings; however, the severity of wasp stings and its clinical course may not be shown immediately at presentation. In China, most wasp stings occur in rural areas [12]. Primary medical institutions lack adequate knowledge of the severity of wasp stings, which may delay appropriate treatment decisions for these patients. Therefore, a simple and easy-to-understand tool combining clinical and biochemical parameters may assist physicians in instituting appropriate management and predicting outcomes. Sequential Organ Failure Assessment (SOFA) and Acute Physiology and Chronic Health Evaluation (APACHEII) scores have been used to classify the severity of wasp sting patients [9,10,14]. However, as these systems were designed to identify patients at risk of deterioration in the ICU, they were unable to identify patients with severe wasp stings at an early stage. In the “Chinese expert consensus on standardized diagnosis and treatment of wasp stings (CECC)”, published in 2018, a guide to classifying the severity of wasp stings was described, but it has not been widely used [12]. In Europe, the poisoning severity score (PSS) was used to assess the severity of poisoning patients (including environmental toxins) [15]. Stays for observation, hospitalization, admission to ICU or general ward and nursing grade were decided according to the assessment results [16]. However, to the author’s knowledge, no previous study has specifically addressed the use of PSS to assess the severity of wasp stings in patients. Therefore, we conducted this study to evaluate the usefulness of PSS as an early prognostic indicator for short-term outcomes in a cohort of 363 patients with wasp stings at Suining Central Hospital from January 2016 to December 2018. Additionally, our study also aimed to explore the clinical characteristics and risk factors associated with mortality. Identifying these factors could play a vital role in effectively identifying high-risk patients and optimizing their management. Ultimately, this knowledge has the potential to contribute to reducing the fatality rate associated with wasp stings.

## 2. Materials and Methods

### 2.1. Ethics Statement

This retrospective study was approved by the IRB (Institutional Review Board) of Suining Central Hospital (Approval No. LLSNCH20200022). Considering the nature of the retrospective study design to review the medical records of patients who completed the treatment, IRB waived the requirement to obtain informed consent. All procedures that involved human participants were conducted in accordance with the ethical standards of the institutional and/or national research committees and compliance with the 1964 Declaration of Helsinki and its later amendments or other comparable ethical standards.

### 2.2. Research Subjects

Due to the implementation of the home quarantine policy in response to the COVID-19 pandemic, there has been a notable decrease in the number of individuals seeking medical attention for wasp stings. To accurately represent the pre-COVID-19 circumstances, our study focused on data collected prior to the implementation of this policy. Specifically, we conducted a retrospective study involving patients with wasp stings who sought treatment at the nephrology department and ICU of Suining Central Hospital in Sichuan Province, China, from January 2016 to December 2018. Suining Central Hospital, located in the interior regions of Sichuan Province, is the sole tertiary grade A general hospital in this area. With an annual intake of over 94,000 hospitalized patients, the hospital typically treats more than 100 cases of wasp stings each year.

The inclusion criteria were: (1) patients with a definite diagnosis of wasp stings; (2) age ≥ 14 years old; (3) the clinical data were complete. The exclusion criteria were as follows: (1) age < 14 years; (2) re-hospitalized patients with wasp stings; (3) wasp sting patients who died before admission; (4) asymptomatic patients; (5) patients who refuse to be admitted to the hospital; (6) patients dead on arrival. We categorized them into survival group (*n* = 349) and death group (*n* = 14) according to the state of discharge.

### 2.3. Definitions

Hypotension was defined as a systolic blood pressure below 90 mmHg or a diastolic blood pressure below 60 mmHg. Acute kidney injury (AKI) was defined based on the following criteria: a rapid increase in serum creatinine levels, indicated by an increase of ≥0.3 mg/dL (26.5 µmol/L) within 48 h compared to baseline, or an increase in creatinine level to 1.5 times or more of the baseline value. Decreased urine output was defined as urine output less than 0.5 mL/kg/h for at least 6 consecutive hours during the oliguric phase [17]. Rhabdomyolysis was defined as serum creatine kinase (CK) level > 1000 U/L or at least 5 times the upper limit of normal [18]. Coagulation abnormalities were determined by the following criteria: activated partial thromboplastin time (aPTT) exceeding the upper limit of normal or prothrombin time (PT) exceeding the upper limit of normal. Liver damage was assessed by elevated levels of alanine aminotransferase (ALT) or aspartate aminotransferase (AST), exceedingly the upper limit of normal values. Hemolysis can be described concisely as follows: the presence of clinical signs and symptoms consistent with hemolysis, such as anemia, jaundice, and/or dark urine. Laboratory evidence of red blood cell destruction, including increased levels of indirect bilirubin and lactate dehydrogenase (LDH) [19].

### 2.4. Clinical Data Collection

Following the approval of the IRB application, we collected the patients’ data from medical records. We collected information on the patients’ demographics (age, gender), the time interval between sting and admission (admission time), number of stings, signs and symptoms (allergic rash, hypotension, macroscopic hematuria, and oliguria or anuria), severe complications (rhabdomyolysis, acute kidney injury (AKI), coagulation disorders, hemolysis, liver dysfunction, acute respiratory distress syndrome (ARDS) and multiple organ dysfunction syndrome (MODS)), inpatient days, and short-term outcomes (death or survival).

We recorded laboratory data on admission, including white blood cells (WBC), activated partial thromboplastin time (APTT), prothrombin time (PT), alanine aminotransferase (ALT), aspartate aminotransferase (AST), indirect bilirubin (I-BIL), creatine kinase (CK), lactate dehydrogenase (LDH), and serum creatinine (SCr).

### 2.5. At Admission, PSS and CECC Were Used as the Criterion for Severity Classification Respectively

The classification of patients using the PSS and CECC was done retrospectively upon admission. The symptoms and signs of the patients were obtained from their medical records, and trained professionals performed the evaluation using the PSS and CECC criteria.

The aim of the PSS is to provide a standardized assessment of the severity of poisoning based on clinical manifestations for research and clinical purposes. It is important to note that the PSS grading system solely considers the observed clinical symptoms and signs and does not take into account factors such as the amount ingested or serum concentrations of the toxic agent. The poisoning severity score (PSS) classifies patients into different severity levels based on observed clinical symptoms and signs related to poisoning. The classification is as follows: (0) none: no symptoms or signs related to poisoning; (1) minor: mild, transient, and spontaneously resolving symptoms; (2) moderate: pronounced or prolonged symptoms; (3) severe: severe or life-threatening symptoms; and (4) fatal poisoning: death. Patients only needed to meet one or more of the criteria to be classified accordingly [20]. We excluded patients with no symptoms and those who died before admission from the analysis, as per our admission criteria, and we categorized the patients into different severity levels, namely minor, moderate, and severe poisoning (Please find the PSS criteria in Supplemental Table S1).

The CECC grades severity as: (1) minor: the number of stings was less than 10, with only local allergic reactions and no organ function involvement; (2) moderate: the number of stings was between 10 and 30, the allergic reaction was classified as Ⅰ-Ⅱ, only 1 organ was involved, sequential organ failure score (SOFA) ≥ 2 points, there was macroscopic hematuria in an early stage; (3) severe: the number of stings was greater than 30, allergic reaction was classified as Ⅲ-Ⅳ or at least 2 organs were involved, SOFA ≥ 2 points for each organ [12]. Patients only needed to meet one or more of the criteria to be classified accordingly (please find the CECC criteria in Supplemental Materials Table S2).

### 2.6. Therapeutic Schedule

During pre-hospital first aid, hydration with 0.9% sodium chloride and glucocorticoid or epinephrine was used for anaphylaxis. After admission, the number of wasp stings was carefully documented, as wasps do not typically leave their stings on the skin.

Treatment schedule for rhabdomyolysis: 0.9% sodium chloride for hydration and sodium bicarbonate to alkalize the urine. Diuretics were given on a hydration-based basis when CK exceeds 1000 µ/L, with the therapeutic goal of achieving urine excretion of at least 2 mL/kg/h [21].

Treatment schedule for AKI: intravenous glucocorticoids were administered (Methylprednisolone 40 mg/d, intravenous drip) for 3–5 days, and the dose was gradually reduced and discontinued for 7–10 days. Hydration and diuresis were performed on patients without oliguria or anuria to achieve at least 100–200 mL/h urine excretion.

Hemodialysis treatment: when wasps sting patients with macroscopic hematuria or AKI (stage Ⅱ and stage Ⅲ), and in some patients in which CK exceeds 10,000 µ/L but without AKI.

There is no dispute about the indications of glucocorticoids in wasp stings [12]. Our patients received glucocorticoid therapy (Methylprednisolone 40 mg/d, intravenous drip) for 3–5 days in the case of macroscopic hematuria, AKI or allergic reaction, and the dose was gradually reduced, and the drug was stopped for 7–10 days.

### 2.7. Statistical Analysis

Continuous variables with normal distribution were expressed as means and standard deviations. Categorical variables without normal distribution were expressed as medians and interquartile ranges. The variables of the two groups were compared by the Mann–Whitney U test. Spearman analysis was performed for the correlation between PSS and CECC. ROC curve analysis of PSS and CECC, respectively, was performed, and Z-test was used to analyze the difference in AUC between them. Univariate and multivariate logistics regression were used to analyze the risk factors of death in wasp sting patients. Receiver operating characteristic (ROC) curves were plotted, and the areas under the ROC curve were calculated. A *p*-value less than 0.05 was considered statistically significant.

## 3. Results

### 3.1. Descriptive Results

**Figure 1:**
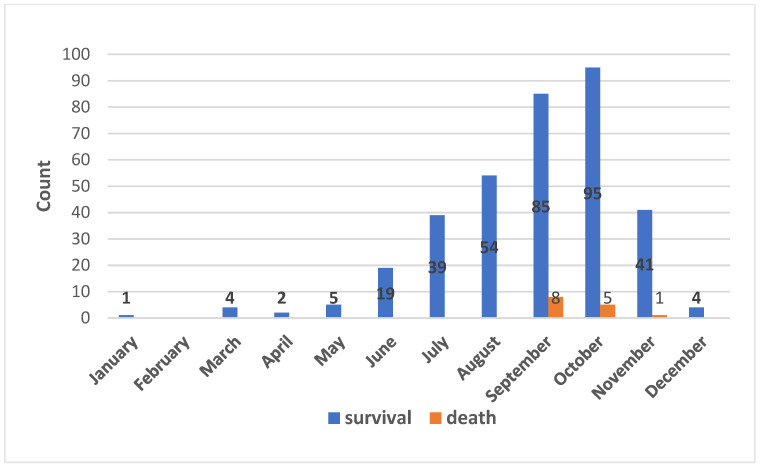
Monthly distribution of wasp sting patients.

Between January 2016 and December 2018, a total of 390 patients were identified with wasp stings. Of these patients, 27 patients were excluded, including three who died on admission and 24 who refused to be hospitalized. Finally, 363 patients were included in this study, which included 219 (60.3%) males and 144 (39.7%) females. The mean age was 55.9 ± 16.3 years. Fourteen (3.9%) patients died during hospitalization, including one in (PSS) grade 1, nine in (PSS) grade 2, and four in (PSS) grade 3. Over the three-year period of this study, the deaths only occurred from September to November (Table 1, Figure 1).

### 3.2. Comparison of Clinical Data between the Survival and the Death Group

The death group had a greater proportion of females (85.7% vs. 37.8%, *p* < 0.001) and was significantly older (71.1 ± 9.8 years vs. 55.3 ± 16.2 years, *p* < 0.001) than that of the survival group. The number of stings (30 vs. 8, *p* < 0.001) and the time from stings to admission (7 h vs. 3 h, *p* = 0.004) in the death group were higher than those in the survival group (*p* < 0.001). The PSS of the death group was significantly higher than that of the survival group (2 vs. 1, *p* < 0.001). The length of hospital stay was significantly shorter in the death group (1 day vs. 4 days, *p* < 0.001) (Table 1).

### 3.3. Comparisons of Complications between the Survival and the Death Group

No allergic rash was seen in the death group (0% vs. 14.3%, *p* = 0.232). One patient in the death group developed hypotension compared to 19 in the survival group (*p* = 0.554). The incidences of rhabdomyolysis, hemolysis, liver dysfunction, coagulation disorder, ARDS, MODS, oliguria (or anuria) and macroscopic hematuria in the death group were significantly higher than those in the survival group (*p* < 0.001). Seven (1.9%) patients were admitted to ICU, including three in the death group and four in the survival group. The incidence of AKI in the death group was significantly higher (78.6%) than that in the survival group (8%) (*p* < 0.001). A larger proportion of patients in the death group (ten patients (71.4%)) received hemodialysis compared to that in the survival group (35 patients (10%)) (*p* < 0.001) (Table 1).

### 3.4. Comparisons of Biochemical Parameters between the Survival and the Death Group

In the first 24 h of hospital admission, the laboratory parameters, including WBC, APTT, PT, ALT, AST, IBIL, CK, LDH, LDH, and SCr values in the death group were significantly higher than those in the survival group (*p* < 0.05) (Table 2).

### 3.5. PSS and CECC

The median PSS was one (1,1) upon admission. Grade 1 (minor) PSS upon admission was seen in 290 (79.9%) cases, grade 2 (moderate) in 59 (16.2%) cases and grade 3 (severe) in 14 (3.9%) cases (Table 3).

Regarding the CECC assessment upon admission, a minor condition was found in 131 (36.1%) cases, moderate in 176 (48.5%) cases, and severe in 56 (15.4%) cases (Table 3).

### 3.6. Spearman Analysis between PSS and CECC

The Spearman analysis showed the correlation between PSS and CECC (r = 0.435, *p* < 0.001), which indicated that there was a correlation between PSS and CECC in assessing the severity of wasp sting patients (Table 3).

### 3.7. ROC Curve Analysis of PSS and CECC

**Figure 2:**
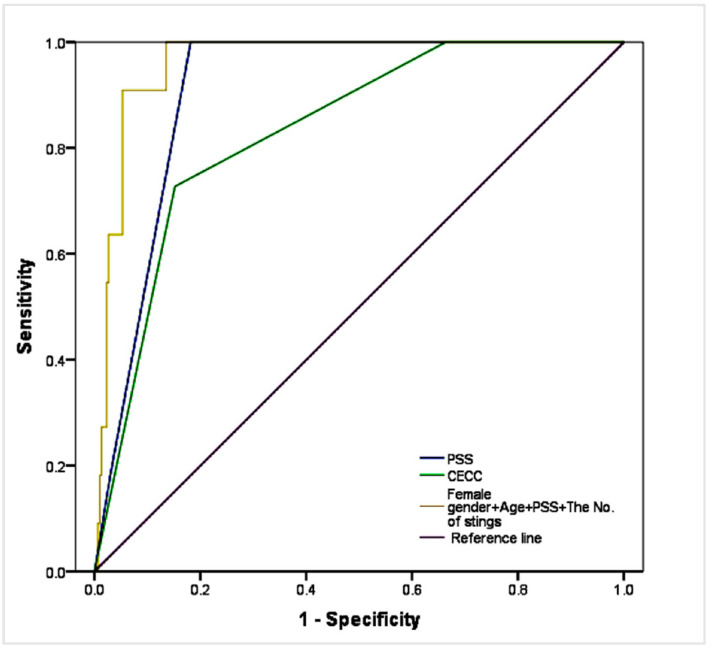
ROC curve analysis for mortality prediction using PSS, CECC, and a composite of four indices (female gender, age, PSS, and number of stings).

The AUC of the PSS and CECC in predicting death in wasp sting patients was 0.890 (95% C.I. 0.806–0.974) and 0.845 (95% C.I. 0.756–0.934), respectively, and they showed a certain predictive power. Although the AUC of the PSS was better than that of CECC, the difference between them was not statistically significant (Z = 0.7230, *p* = 0.4697). Table 4 shows the optimal cut-off value, sensitivity, specificity, positive predictive value, and negative predictive values of PSS and CECC (Figure 2, Table 4).

### 3.8. Univariate Logistic Regression Analysis

Table 5 presents the results of the univariate logistic regression analysis, which aimed to identify the risk factors associated with death in patients with wasp stings. Considering our pre-specified hypothesis that the month of occurrence could be a risk factor for death, as deaths exclusively occur between September and November. We compared various variables between the death group and the survival group. The analysis revealed significant statistical differences in age, gender, number of stings, admission time, PSS, and biochemical parameters. Considering that the PSS encompasses multiple systems, including cardiovascular, respiratory, liver, kidney, and muscular, we considered age, gender, number of stings, admission time, PSS, and month as independent variables in the subsequent univariate logistic regression analysis. The findings of the univariate analysis demonstrated a significant association between death in wasp sting patients and several previously identified predictors: month and number of stings. Furthermore, we uncovered novel potential predictors, namely age, female gender, admission time, and PSS. These six variables were then included in the multivariable model for further investigation.

### 3.9. Multivariate Logistic Regression Analysis

In order to identify the independent risk factors for death in patients with wasp stings, we performed a backward multivariate logistic regression analysis. The results highlighted that female gender, older age, higher number of stings, and elevated PSS were all determined as independent risk factors for death. These factors exhibited corresponding odds ratios of 8.651, 1.103, 1.033, and 6.768, respectively (Table 6). We conducted diagnostic tests, including variance inflation factor (VIF) analysis, to assess whether multicollinearity existed among the independent variables. The outcomes indicated no significant multicollinearity among the variables included in our analysis.

### 3.10. ROC Curve Analysis of Death Risk Factors

Following the principle of the Youden Index, we selected the optimal cut-off values that maximize sensitivity and specificity on the ROC curve. Table 7 presents the results of the ROC analysis for these four risk factors, along with the corresponding cut-off values.

These four indexes of female gender, age, PSS and the number of stings were combined as prediction criteria; the prediction probability saved in the multivariate logistics regression analysis was taken as independent variable, and death as dependent variable. The ROC curve analysis showed that AUC = 0.962 (95% C.I. 0.936–0.988, *p* < 0.001) (Figure 2).

## 4. Discussion

Among hymenopteran stings, encounters with wasps are associated with the highest incidence of adverse clinical outcomes [4,5,22]. Notably, wasp stings have been identified as a leading cause of community-acquired acute kidney injury (AKI) in Asia [23,24]. This study aimed to investigate the clinical characteristics and risk factors associated with mortality in patients who suffered from wasp stings. We identified several early warning signs that were significantly associated with death, including being female, older age (age > 61), a higher number of stings (No. > 29), and a higher poisoning severity score (PSS) level (PSS > 1). These findings provide valuable insights into the management and prognosis of patients affected by wasp stings.

Firstly, our study revealed a higher mortality rate among patients with wasp stings, with 3.9% of patients succumbing to their injuries. This highlights the severity of such incidents and underscores the need for prompt medical attention. Wasp stings and related deaths show a seasonal pattern [25,26]. In our cohort, 79.6% of wasp stings occurred from August to November, and 14 fatal cases were only reported between September and November. This seasonality is related to the habits of the wasps, as they build their nests and mate during autumn, which increases the likelihood of human encounters and subsequent attacks [27]. To address this issue, the government and the media should increase public awareness of the hazards posed by wasps, particularly during late summer and fall when outdoor activities and farming are more common.

Our analysis of demographic data revealed age and gender as significant risk factors for mortality. The death group had a significantly higher mean age compared to the survival group (71.1 ± 9.8 years vs. 55.3 ± 16.2 years), indicating that older individuals are more vulnerable to complications resulting from wasp stings. Moreover, a higher proportion of females were observed in the death group (85.7% vs. 14.3% male), implying that gender may play a role in the severity of reactions to wasp venom. This could be attributed to factors such as women’s involvement in agricultural activities, particularly in rural areas where industrialization is limited, or it may be due to their older age and higher prevalence of underlying medical conditions. Further investigations are needed to explore the underlying mechanisms.

The severity of the reaction was also associated with the number of stings. Previous studies by Xie et al. and Liu et al. have shown that the overall incidence of severe complications is higher in patients with more than 10 stings [5,28]. In our study, the number of stings was identified as a risk factor for mortality, with more than 29 stings being associated with increased risk. It is important to note, however, that the number of stings does not necessarily correlate with the severity of anaphylaxis [29,30,31]. Patients in the death group experienced a larger number of stings and received delayed medical intervention compared to the survival group. These findings highlight the importance of immediate medical care and the need for public education regarding the prompt recognition and treatment of wasp stings.

The severity of systemic complications was significantly higher in the death group. The death group exhibited higher incidences of rhabdomyolysis, hemolysis, liver dysfunction, coagulation disorders, acute respiratory distress syndrome (ARDS), multiple organ dysfunction syndrome (MODS), oliguria (or anuria), and macroscopic hematuria. These complications likely contribute to the poor outcomes observed in these patients. Early recognition and appropriate management of these complications are crucial for improving patient survival [12]. Serious complications such as rhabdomyolysis, hemolysis, acute kidney injury (AKI), and abnormal coagulation require laboratory tests for confirmation. Within 24 h of admission, patients in the death group had significantly abnormal laboratory test results compared to those in the survival group. Higher levels of transaminases, bilirubin, lactate dehydrogenase, leukocytes, and serum creatinine indicated more severe complications such as liver damage, rhabdomyolysis, inflammatory reactions, and acute kidney injury among patients in the death group. Therefore, it is recommended to perform timely laboratory tests, including blood routine, liver and kidney function, clotting function, and myocardial enzyme analysis, after being stung by a swarm of wasps to detect possible serious complications.

The Chinese Society of Toxicology published a consensus statement on the standardized diagnosis and treatment of wasp stings (CECC) in 2018 [12]. However, the wider application of this consensus criterion is likely to be limited due to its complex evaluation criteria. Mong et al. reported the use of the poisoning severity score (PSS) to assess the severity of patients, including those with wasp stings, in the emergency department [16]. Our study demonstrated a correlation between PSS and CECC in assessing the severity of wasp sting patients. However, further investigation is required to understand the factors contributing to the lower than expected correlation between PSS and CECC. Both scoring systems showed predictive power for mortality, as indicated by the area under the curve (AUC) values obtained through ROC curve analysis. Although the AUC of PSS was slightly better than that of CECC, the difference was not statistically significant. These findings suggest that both scoring systems can serve as reliable tools for risk stratification and prognostic assessment in clinical practice.

Furthermore, our univariate and multivariate logistic regression analyses identified several independent risk factors for mortality in patients with wasp stings. Female gender, advanced age, a higher number of stings, and a higher PSS were consistently associated with an increased risk of mortality. These findings highlight the importance of considering these factors when evaluating patients and designing targeted therapeutic interventions.

Overall, our study provides novel insights into the clinical characteristics and risk factors associated with mortality in patients with wasp stings. The results emphasize the need for early medical intervention, particularly among elderly female individuals and those with a higher number of stings or a higher PSS level. Additionally, our findings support the use of both PSS and CECC as reliable tools for assessing disease severity and predicting patients’ outcomes. Further research is warranted to validate our findings and explore potential therapeutic strategies to improve the prognosis of patients affected by wasp stings.

## 5. Limitations of this Study

Although our study provides valuable insights into the clinical manifestations and risk factors associated with mortality following swarm wasp stings, we acknowledge several limitations. Firstly, the small number of events prevented us from conducting a detailed statistical analysis, limiting the generalizability of our findings. Secondly, as a retrospective study conducted at a single center, there is a possibility of selection bias and confounding factors that may have influenced our results. Therefore, caution should be exercised when extrapolating these findings to other populations, including the general Chinese population. Thirdly, our prediction model only considers relevant clinical data at admission and does not account for the impact of previous diseases and treatments on prognosis. Fourth, the medical records do not indicate whether patients stung by wasps came from rural or urban areas. Additionally, we did not consider the effect of body mass index (BMI) on the toxin density of wasps, which is another limitation. Furthermore, our study lacks information on the specific species of wasps responsible for the stings. Future research should involve multicenter prospective studies to validate and further expand upon our findings.

## 6. Conclusions

This study highlights the utility of the poisoning severity score (PSS) as an early prognostic tool for patients with wasp stings. The use of the PSS as a predictive tool may assist clinicians in making timely decisions regarding patient care and interventions. Importantly, our study identifies specific early warning signs associated with mortality, including female gender, increased age (>61), higher number of stings (>29), and higher PSS levels (>1). The overall mortality rate in our study was 3.9%, and it is noteworthy that all deaths occurred within the first three days of hospital admission, particularly during the late summer and fall months. These findings emphasize the necessity for heightened vigilance and close monitoring of patients during this period. Although our study provides significant insights, further research and validation studies are required to confirm the effectiveness and generalizability of the PSS as a prognostic tool for patients with wasp stings. Such studies will contribute to the continued refinement and application of the PSS in clinical practice.

## Figures and Tables

**Table 1:** Comparison of clinical data between the survival and the death group.

Variable	Survival Group (*n* = 349)	Death Group (*n* = 14)	*p*
Age (years)	55.3 ± 16.2	71.1 ± 9.8	<0.001
Gender (M:F)	217:132	2:12	<0.001
Number of stings	8 (4, 15)	30 (20, 45)	<0.001
Admission time (*h*)	3 (2, 5)	7 (3.7, 10)	0.004
Poisoning severity score	1 (1, 1)	2 (2, 3)	<0.001
Grade 1 (*n*)	289	1	
Grade 2 (*n*)	50	9	
Grade 3 (*n*)	10	4	
Inpatient days (day)	4 (3,7)	1 (1, 2.2)	<0.001
Allergic rash *n* (%)	50 (14.3)	0 (0)	0.232
Hypotension *n* (%)	19 (5.4)	1 (7.1)	0.554
AKI * *n* (%)	28 (8%)	11 (78.6)	<0.001
Rhabdomyolysis *n* (%)	92 (26.4)	13 (92.9)	<0.001
Hemolysis *n* (%)	49 (14)	12 (85.7)	<0.001
Oliguria or anuria *n* (%)	18 (5.2)	9 (64.3)	<0.001
Macroscopic hematuria *n* (%)	38 (10.9)	13 (92.9)	<0.001
Coagulation abnormalities *n* (%)	126 (36.1)	13 (92.9)	<0.001
Liver damage *n* (%)	69 (19.8)	13 (92.9)	<0.001
Dialysis *n* (%)	35 (10)	10 (71.4)	<0.001
MODS ** *n* (%)	43 (12.3)	13 (92.9)	<0.001
ARDS ^#^ *n* (%)	4 (1.1)	9 (64.3)	<0.001
ICU ^##^ *n* (%)	4 (1.1)	3 (21.4)	<0.001

* Acute kidney injury, ** multiple organ dysfunction syndrome, ^#^ acute respiratory distress syndrome, ^##^ Intensive care unit.

**Table 2:** Comparison of biochemical parameters between the survival and the death group.

Biochemical Parameters	Survival Group (*n* = 349)	Death Group (*n* = 14)	*p*
SCr * (59–104 µmol/L)	66 (55, 77)	78 (68.5, 140)	0.004
CK ** (40–200 U/L)	199 (117.5, 415.5)	2321 (407.7, 5342.5)	<0.001
AST *** (13–35 U/L)	35.5 (26, 55.2)	557 (255, 1505.5)	<0.001
IBIL **** (0–18 µmol/L)	8.1 (5.2, 16.8)	39.4 (19.8, 83.4)	<0.001
ALT ^#^ (7–40 U/L)	22 (17, 37)	286 (58.5, 1369.5)	<0.001
PT ^##^ (11–14.5 s)	13.7 (13, 14.6)	15 (14, 17)	0.002
APTT ^###^ (26–40 s)	51.3 (36.8, 91.2)	120.5 (94.1, 180)	<0.001
LDH (120–250 U/L)	215 (179, 277.5)	1671 (1195.5, 2795)	<0.001
WBC ^####^ (3.5–9.5 × 10^9^/L)	11.8 (8.4, 15.8)	26.3 (21.2, 33.6)	<0.001

* Serum creatinine, ** creatine kinase, *** aspartate aminotransferase, **** indirect bilirubin, ^#^ alanine transaminase, ^##^ prothrombin time, ^###^ activated partial thromboplastin time, ^####^ white blood cells.

**Table 3:** Spearman analysis between PSS and CECC.

		PSS			Sum
		Grade 1	Grade 2	Grade 3	
**CECC**	Minor	124	7	0	131
	Moderate	147	26	3	176
	Severe	19	26	11	56
Sum		290	59	14	363

Spearman analysis, r = 0.435, *p* < 0.001.

**Table 4:** The ROC analysis of PSS and CECC in the optimal cut-off scores.

Score	Value				The Comparison of AUC		
	Sensitivity (%)	Specificity (%)	+PV (%)	−PV (%)	AUC (95% C.I.)	Z	*p*
PSS > 1	92.86	82.81	17.8	99.7	0.890 (0.806–0.974)	0.723	0.469
CECC > 2	71.43	86.82	17.9	98.7	0.845 (0.756–0.934)		

+PV: positive predictive value; −PV: negative predictive value.

**Table 5:** Univariate logistic regression analysis of risk factors of death.

Variable	β	Wald χ^2^	*p*	*OR*	95% C.I.
Age	0.102	12.099	0.001	1.108	1.046–1.173
Female	2.289	8.797	0.003	9.864	2.174–44.762
Admission time	0.004	0.071	0.790	1.004	0.974–1.035
Month	1.694	5.759	0.016	5.440	1.364–21.699
Number of stings	0.043	14.808	<0.001	1.044	1.022–1.068
PSS *	2.135	29.403	<0.001	8.460	3.910–18.305

* Poisoning severity score.

**Table 6:** Multivariate logistic regression analysis of risk factors of death.

Variable	β	Wald χ^2^	*p*	OR	95% C.I.	*VIF*
Age	0.098	5.029	0.025	1.103	1.012–1.201	1.160
Female	2.158	4.885	0.027	8.651	1.277–58.629	1.048
Number of stings	0.033	4.852	0.028	1.033	1.004–1.064	1.209
PSS *	1.912	9.306	0.002	6.768	1.981–23.120	1.297

Hosmer–Lemeshow analysis: χ^2^ = 0.826 *p* = 0.999, * poisoning severity score.

**Table 7:** The ROC analysis of these four risk factors.

Variable	Cut-off	Sensitivity (%)	Specificity (%)	AUC (95% C.I.)
Gender	Female	81.8	62.7	0.723 (0.624–0.855)
Age	>61	90.9	56.1	0.865 (0.690–0.896)
Number of stings	>29	72.7	89.4	0.865 (0.753–0.976)
PSS *	>1	92.9	82.8	0.890 (0.806–0.974)

* Poisoning severity score.

## Data Availability

All data and materials were obtained from Suining Central Hospital.

## Supplementary Materials

The following supporting information can be downloaded at: https://www.mdpi.com/article/10.3390/jcm12185789/s1, Table S1: The criteria of the poisoning severity score (PSS); Table S2: Criteria of the Chinese Expert Consensus on Standardized Diagnosis and Treatment of Wasp Stings (CECC).

## Abbreviations

PSS: poisoning severity score; CECC: Chinese expert consensus on standardized diagnosis and treatment of wasp stings; AKI: acute kidney injury; ARDS: acute respiratory distress syndrome; MODS: multiple organ dysfunction syndrome; WBC: white blood cells; APTT: activated partial thromboplastin time; PT: prothrombin time; ALT: alanine aminotransferase; AST: aspartate aminotransferase; I-BIL: indirect bilirubin; CK: creatine kinase; LDH: lactate dehydrogenase; SCr: the serum creatinine; SOFA: Sequential Organ Failure Assessment; APACHEII: Acute Physiology and Chronic Health Evaluation.
