# Supplementary material for: Clinical Manifestations and Risk Factors Associated with 14 Deaths following Swarm Wasp Stings in a Chinese Tertiary Grade A General Hospital: A Retrospective Database Analysis Study
Source: J Clin Med. 2023 Sep 6;12(18):5789. doi: 10.3390/jcm12185789 (PMC10532120; doi:10.3390/jcm12185789)
Supplement: Supplementary file 1 [file jcm-12-05789-s001.zip › jcm-2435944-supplementary.pdf]

Supplemental Materials

Table S1. The criteria of the poisoning severity score (PSS)

Organ	None	Minor	Moderate	Severe	Fatal
	0	1	2	3	4
	No symptoms or signs	Mild transient, and spontaneously resolving symptoms	Pronounced or prolonged signs or symptoms	Severe or life-threatening	Death
Cardiovascular system		Isolated extrasystoles	Bradycardia (HR 40-50 in adults) Tachycardia (HR140-180 in adults), Chest pain, Conductance, disturbance, Hypertension, Hypotension	Bradycardia (HR < 40 for adults) Tachycardia (HR > 180 for adults) Cardiac arrest	
Respiratory system		Irritation, coughing, breathlessness, mild dyspnea, mild bronchospasm Chest X-ray: abnormal with minor or no symptoms	Prolonged coughing, bronchospasm, dyspnea, stridor, hypoxemia requiring extra oxygen Chest X-ray: abnormal with moderate symptoms	Manifest respiratory insufficiency airway obstruction, pulmonary edema, ARDS*, pneumonitis Chest X-ray: abnormal with severe symptoms	
Nervous system		Vertigo, tinnitus, ataxia, Mild extrapyramidal symptoms, Paresthesia	Unconsciousness with appropriate response to pain, Confusion, agitation, hallucinations, delirium, Infrequent, generalized, or local seizures	Deep coma unresponsive to pain Extreme agitation Generalized seizures, status epilepticus Massive hemorrhage, perforation	
GI tract		Vomiting, diarrhea, pain	Pronounced or prolonged vomiting, diarrhea, pain ileus, Dysphagia	Severe dysphagia	
Metabolic imbalance		Mild acid-based disturbances, Mild electrolyte and fluid disturbances, Mild hypoglycemia	More pronounced acid-based disturbances, more pronounced electrolyte and fluid disturbances, more pronounced hypoglycemia	Severe acid-based disturbances, Severe electrolyte and fluid disturbances, Severe hypoglycemia	
Liver		Minimal rise in serum enzymes	Rise in serum enzymes no diagnostic bio-chemical or clinical evidence of liver dysfunction	Rise in serum enzymes biochemical or clinical evidence of liver dysfunction	
Kidney		Minimal proteinuria/hematuria	Massive proteinuria/hematuria, Renal dysfunction	Renal failure	
Muscular		Mild pain, tenderness	Pain, rigidity, cramping, fasciculations, Rhabdomyolysis	Intense pain, extreme rigidity, extensive cramping, fasciculations, Rhabdomyolysis with complications	
Local effects on skin		Irritation,1st degree burns	2nd degree burns in 10%-50% of body surface or 3rd degree burns in <2% of body	2nd degree burns in >50% of body surface or 3rd degree burns	
Local effects on eye		Irritation, redness, lacrimation, mild palpebral edema	Intense irritation, corneal abrasion Minor(punctate) corneal ulcers	Corneal ulcers (other than punctate), perforation	

* Acute Respiratory Distress Syndrome, Patients only need to meet one or more of the criteria to be classified accordingly

Table S2. Criteria of the Chinese Expert Consensus on Standardized Diagnosis and Treatment of Wasp Stings (CECC)

No. of stings	<10	10-30	>30
Allergic reactions **	local anaphylaxis	stage Ⅰ ~ Ⅱ	stage Ⅲ ~ Ⅳ
Involvement of organs	zero	one	two or more
SOFA***	-	≥2points	≥2 points per system (6 systems)
Visible hematuria	-	in the early stage	-
Result	Mild	Moderate	Severe

**The severity of allergic reactions is assessed according to the Ring and Messmer standards. ***sequential organ failure score. Patients only need to meet one or more of the criteria to be classified accordingly
